# Safety, antitumor activity, and pharmacokinetics of dostarlimab, an anti-PD-1, in patients with advanced solid tumors: a dose–escalation phase 1 trial

**DOI:** 10.1007/s00280-021-04358-3

**Published:** 2021-11-08

**Authors:** Amita Patnaik, Glen J. Weiss, Drew W. Rasco, Lisa Blaydorn, Amy Mirabella, Murali Beeram, Wei Guo, Sharon Lu, Hadi Danaee, Kristen McEachern, Ellie Im, Jasgit C. Sachdev

**Affiliations:** 1 South Texas Accelerated Research Therapeutics, San Antonio, TX USA; 2 Western Regional Medical Center, Cancer Treatment Centers of America, Goodyear, AZ USA; 3 HonorHealth Research Institute/Translational Genomics Research Institute (TGen), Scottsdale, AZ USA; 4 GlaxoSmithKline, Waltham, MA USA; 5 Ribon Therapeutics, Cambridge, MA USA; 6 Present Address: SOTIO, LLC, Cambridge, MA USA; 7 Present Address: Blueprint Medicines, Cambridge, MA USA

**Keywords:** Advanced cancer, Programmed cell death receptor 1, Dostarlimab, TSR-042, Phase 1 clinical trial

## Abstract

**Purpose:**

New immuno-oncology therapies targeting programmed cell death receptor 1 (PD-1) have improved patient outcomes in a broad range of cancers. The objective of this analysis was to evaluate the PK, pharmacodynamics (PDy), and safety of dostarlimab monotherapy in adult patients with previously-treated advanced solid tumors who participated in parts 1 and 2A of the phase 1 GARNET study.

**Methods:**

Part 1 featured a 3 + 3 weight-based dose–escalation study, in which 21 patients received dostarlimab 1, 3, or 10 mg/kg intravenously every 2 weeks. The 2 fixed-dose nonweight-based dosing regimens of dostarlimab 500 mg every 3 weeks (Q3W) and 1000 mg every 6 weeks (Q6W) were evaluated using a modified 6 + 6 design in part 2A (*n* = 13). In parts 1 and 2A, treatment with dostarlimab could continue for up to 2 years or until progression, unacceptable toxicity, patient withdrawal, investigator’s decision, or death.

**Results:**

The dostarlimab PK profile was dose proportional, and maximal achievable receptor occupancy (RO) was observed at all dose levels in the weight-based and fixed-dose cohorts. Trough dostarlimab concentration after administration of dostarlimab 500 mg Q3W was similar to that after dostarlimab 1000 mg Q6W, the values of which (≈40 µg/mL) projected well above the lowest dostarlimab concentration required for full peripheral RO. No dose-limiting toxicities were observed.

**Conclusions:**

Dostarlimab demonstrated consistent and predictable PK and associated PDy. The observed safety profile was acceptable and characteristic of the anti-PD-1 drug class.

**Trial registration:** ClinicalTrials.gov, NCT02715284. Registration date: March 9, 2016.

**Supplementary Information:**

The online version contains supplementary material available at 10.1007/s00280-021-04358-3.

## Background

Programmed cell death 1 (PD-1) is an immune checkpoint receptor expressed on antigen-activated and exhausted T cells that delivers inhibitory signals to control local inflammatory responses and maintain self-tolerance [1, 2]. PD-1 receptor binding by tumor-expressed ligands PD-L1 and PD-L2 inhibits T-cell proliferation and cytokine production [1, 2]. Upregulation of PD-L1 by many tumors enables subversion of the PD-1/PD-L1 pathway, sufficient to blunt cytotoxic T-cell response within the tumor microenvironment, and has been associated with a poor prognosis [1–4]. Blocking PD-1/PD-L1 binding reverses immune evasion and restores adaptive immune response against the tumor [5, 6]. Furthermore, PD-(L)1 monoclonal antibodies (mAbs) have demonstrated antitumor activity in patients with various solid tumors [7–12].

Dostarlimab (JEMPERLI) is a humanized anti-PD-1 immunoglobulin (Ig) G4 mAb that binds with high affinity to the PD-1 receptor and effectively blocks interaction with PD-1 ligands PD-L1 and PD-L2 [13]. Dostarlimab is approved as a monotherapy in patients with dMMR recurrent or advanced solid tumors that have progressed on or following prior treatment and who have no satisfactory alternative treatment options in the United States (US). It is also approved in adult patients with dMMR (US) or dMMR/MSI-H (European Union) recurrent or advanced EC that has progressed on or following prior treatment with a platinum-containing regimen. [add 3 new references: GlaxoSmithKline. Jemperli. Accessed September 1, 2021. https://gskpro.com/content/dam/global/hcpportal/en_US/Prescribing_Information/Jemperli/pdf/JEMPERLI-PI-MG.PDF; European Medicines Agency. Jemperli. Accessed September 1, 2021. https://www.ema.europa.eu/en/medicines/human/EPAR/jemperli; US Food and Drug Administration. FDA grants accelerated approval to dostarlimab-gxly for dMMR advanced solid tumors. Accessed August 23, 2021. https://www.fda.gov/drugs/resources-information-approved-drugs/fda-grants-accelerated-approval-dostarlimab-gxly-dmmr-advanced-solid-tumors]

Dostarlimab has been tested in a first-in-human phase 1 study (GARNET) designed to assess safety, tolerability, and antitumor activity of monotherapy in patients with advanced solid tumors. The study was conducted in several sequential parts: part 1 consisted of weight-based dose escalation in a 3 + 3 design; part 2A evaluated fixed doses in a modified 6 + 6 design, and part 2B consists of expansion cohorts in selected tumor types. Evaluation of safety and tolerability with weight-based dosing schedules (part 1) and fixed-dose regimens (part 2A) of this ongoing trial (NCT02715284) are complete and are reported herein.

The objectives of this publication are to report pharmacokinetics (PK), pharmacodynamics (PDy), safety, and preliminary antitumor activity of dostarlimab in parts 1 and 2A of GARNET.

## Methods

### Study design

GARNET is a multicenter, open-label, first-in-human, phase 1 study to assess maximum tolerated dose (MTD), recommended treatment dose (RTD), schedule, safety, and preliminary antitumor activity of dostarlimab administered via a 30-min IV infusion in patients with advanced solid tumors. The study was conducted in three stages, a weight-based, dose–escalation stage (part 1), followed by a fixed-dose stage (part 2A) (Supplementary Figure S1), followed by dose expansion cohorts in selected tumor types (part 2B) treated at the recommended therapeutic dose determined in parts 1 and 2A. The primary objective of parts 1 and 2A was to evaluate the safety and tolerability of dostarlimab in patients with all solid tumors and to determine the recommended therapeutic dose and schedule, with secondary objectives to evaluate antitumor activity by immune-related objective response rate and to characterize PK and immunogenicity. The primary objective of part 2B was to evaluate the antitumor activity of dostarlimab in patients with select tumor types, including mismatch repair deficient (dMMR)/microsatellite instability–high (MSI-H) endometrial cancer, MMRp/MSS endometrial cancer, NSCLC, dMMR/MSI-H and POLE-mutated nonendometrial solid tumors, and platinum-resistant ovarian cancer. In all parts, study treatment could continue for up to 2 years or until progression, unacceptable toxicity, patient withdrawal, investigator’s decision, or death.

Part 1 enrolled patients between March and October 2016. Dostarlimab was administered every 2 weeks (Q2W), on days 1 and 15 of each 28-day treatment cycle, at a starting weight-based dose of 1 mg/kg with subsequent dose escalation to levels of 3 and 10 mg/kg per a modified 3 + 3 design. Patients were evaluated for dose-limiting toxicity (DLT) based on the adverse events (AEs) reported during the first cycle (28 days). The dose level was declared safe if < 33% of patients experienced DLT. In addition, 9 patients (3 at the 1 mg/kg dose, and 6 at the 10 mg/kg dose) were enrolled specifically for additional PK/PDy sampling; they did not receive dostarlimab on day 15 of cycle 1, but did subsequently follow a Q2W schedule throughout the rest of the study treatment period.

Part 2A enrolled patients between December 2016 and February 2017. Following completion of part 1, dostarlimab safety and tolerability at fixed-dose schedules of 500 mg every 3 weeks (Q3W) and 1000 mg every 6 weeks (Q6W) were evaluated in patients with advanced solid tumors per a modified 6 + 6 design. Cycle durations and DLT observation periods were 21 days for the Q3W cohort and 42 days for the Q6W cohort. Patients in each cohort received dostarlimab on day 1 of every cycle. Protocol-mandated treatment modifications for both study parts are summarized in Supplementary Tables S1 and S2.

### Patient eligibility

Patients aged 18 years or older with advanced (unresectable) or metastatic solid tumors and disease progression after treatment with available standard of care treatments or treatment intolerance were eligible for enrollment in parts 1 and 2A of GARNET. Patients with previously treated, stable brain metastases were eligible. For full inclusion and exclusion criteria, see Supplementary Table S3.

### Assessments and outcomes

#### Pharmacokinetics and pharmacodynamics

The blood sample regimen is detailed in the Supplementary Methods. Serum dostarlimab concentrations were quantified using enzyme-linked immunosorbent assay (Supplementary Methods).

PK analysis was performed using noncompartmental (WinNonlin Version 8.0, Pharsight, Mountain View, CA) and 2-compartmental analysis methods (NONMEM, ICON Development Solutions, Ellicott City, MD). Maximum (*C*_max_) and minimum (*C*_trough_) serum dostarlimab concentrations and time to *C*_max_ (*T*_max_) were the observed values. Total systemic exposure to dostarlimab was estimated by calculating the area under the serum dostarlimab concentration–time curve (AUC) using the linear trapezoidal method (linear up, log down). Terminal elimination half life was calculated as ln(2)/*k*. Body weight was evaluated as a covariate for dostarlimab clearance before exploring the fixed-dose strategy in part 2A and further evaluating combined data from both parts 1 and 2A. In addition, a preliminary population PK model was developed based on the data available from weight-based doses in 17 patients. The preliminary model was developed to generally describe the PK characteristics of dostarlimab and assess the effect of weight on PK exposures in support of exploring fixed dosing of dostarlimab in upcoming cohorts. Multicompartment mode structures were explored. Given the purpose of the modeling, only body weight was assessed as a covariate for drug clearance. Based on this model, 1000 patients were simulated under dosing regimens of 500 mg Q3W or 1000 mg Q6W. Confidence and prediction intervals around the minimum concentrations at the end of the dosing interval were calculated and reported.

To analyze PDy, the flow cytometry was used to evaluate direct PD-1 receptor occupancy (RO) by dostarlimab on circulating CD3+ peripheral blood mononuclear cells [14, 15]. The extent of functional RO by dostarlimab was further determined by measuring interleukin-2 (IL-2) concentrations after ex vivo stimulation of T cells. Whole blood collected from patients was incubated with superantigen staphylococcal enterotoxin B in the presence of saturating concentrations of dostarlimab or isotype control to stimulate IL-2 production. The ratio of IL-2 from saturating with dostarlimab versus isotype control is a measure of RO of which 1 reflects the maximal stimulation and, therefore, full RO [15].

### Safety

AEs were coded using the Medical Dictionary for Regulatory Activities, version 20.0, and graded according to the National Cancer Institute Common Terminology Criteria for Adverse Events, version 4.03. Predefined DLT criteria for parts 1 and 2A are described in Supplementary Table S4. Patients received appropriate supportive care measures, including prophylactic cytokines (after cycle 1; i.e., granulocyte colony-stimulating factor [G-CSF]), as deemed necessary by the treating investigator and according to current American Society of Clinical Oncology guidelines [16].

### Clinical antitumor activity

In part 1, radiographic evaluation (computed tomography/magnetic resonance imaging of chest, abdomen, and pelvis) and appropriate testing of serum-based tumor markers were conducted at time of screening, every 10 weeks (± 10 days) until month 12, and every 12 weeks (± 10 days) thereafter. In part 2, radiographic evaluations were conducted 12 weeks after receipt of first dostarlimab dose, every 6 weeks (± 10 days) until month 12, then every 12 weeks thereafter. For both parts 1 and 2A, tumor responses were assessed by investigators per immune-related Response Evaluation Criteria in Solid Tumors (irRECIST) [17–19]. Per irRECIST, complete responses (irCRs), partial responses (irPRs), and progressive disease (irPD) were confirmed with a second tumor assessment at least 4 weeks after the first assessment.

### Statistical analysis

Patients who received at least one dose of dostarlimab were included in the description of baseline characteristics and analysis of safety and antitumor activity. PK-related analyses were based on the PK population, defined as all patients who received at least one dose of study drug and had one PK sample collected after start of dosing. Descriptive statistics, including means, medians, ranges, and standard deviations, were calculated. SAS version 9.4 (SAS Institute Inc., Cary, NC) was used for statistical analyses.

## Results

### Patient characteristics and study drug exposure

Patients were enrolled in part 1 from March 25, 2016, to October 12, 2016, and in part 2A from December 14, 2016, to February 17, 2017. Twenty-one patients received dostarlimab in part 1, and 13 patients received dostarlimab in part 2A (Supplementary Table S5; Supplementary Figure S2). As of the data cutoff date (August 10, 2018), 20 of 21 patients in part 1 and all patients in part 2A had discontinued treatment. Baseline characteristics are shown in Supplementary Table S5.

#### Pharmacokinetics and pharmacodynamics

Dostarlimab PK parameters for cycle 1 are presented in Table 1, and multiple-dose PK parameters are presented in Supplementary Table S6. In cycle 1, maximum concentrations were reached shortly after the end of infusion, followed by biexponential decline. Following multiple-dose administration, the characteristics of PK profiles were generally comparable to those in cycle 1. The arithmetic mean concentrations of dostarlimab in parts 1 and 2A increased with increasing dose and remained quantifiable throughout the study.

**Table 1:** Summary statistics for dostarlimab pharmacokinetic parameters, cycle 1 for parts 1 and 2A

		Part 1: Geometric mean (GCV%)								
		AUC_(0-∞)_ (µg·h/mL)	AUC_(0-τ)_ (µg·h/mL)	*C*_max_ (µg/mL)	*t*_max_^a^ (h)	*C*_trough_^b^ (µg/mL)	*t*_1/2_ (d)	*CL* (L/d)	*V*_z_ (L)	*V*_ss_ (L)
1 mg/kg, DLT-eval (*n* = 3)	4440–9570^c^		3417 (31.7)	21.78 (25.6)	2.92 (1.52–2.95)	6.382 (35.9)	8.2–14.2^c^	0.336–0.367^c^	3.97–7.50^c^	4.12–7.29^c^
1 mg/kg, PK/PDy (*n* = 3)	5872 (93.3)		4219 (63.8)	20.43 (17.7)	1.50 (0.55–1.50)	2.622 (148.4)	14.5 (67.1)	0.326 (64.8)	6.84 (7.6)	6.73 (17.8)
3 mg/kg (*n* = 3)	26,630 (9.4)		10,790 (9.7)	66.17 (9.6)	1.52 (1.50–2.95)	23.70 (8.9)	19.1 (23.8)	0.189 (33.5)	5.20 (33.5)	5.11 (32.1)
10 mg/kg, DLT-eval (*n* = 6)	59,830 (26.7)		36,480 (24.4)	228.4 (22.2)	1.52 (1.50–3.07)	60.63 (34.7)	10.2 (35.5)	0.302 (22.3)	4.43 (18.3)	4.33 (19.8)
10 mg/kg, PK/PDy (*n* = 6)	101,900 (35.8)^d^		63,670 (28.2)^d^	251.1 (19.2)	1.50 (1.43–3.12)	53.15 (35.0)^d^	20.5 (18.2)^d^	0.160 (13.6)^d^	4.73 (14.2)^d^	4.57 (14.0)^d^

	Part 2A: Geometric mean (GCV%)								
	AUC_(0-∞)_ (µg·h/mL)	AUC_(0-τ)_^e^ (µg·h/mL)	*C*_max_ (µg/mL)	*t*_max_^a^ (h)	*C*_trough_^f^ (µg/mL)	*t*_1/2_ (d)	*CL* (L/d)	*V*_z_ (L)	*V*_ss_ (L)
500 mg Q3W (*n* = 6)	55,510 (24.2)	35,730 (20.2)	171.1 (20.0)	0.96 (0.50–3.02)	39.17 (26.7)	14.5 (12.3)	0.216 (24.2)	4.51 (20.5)	4.38 (18.0)
1000 mg Q6W (*n* = 7)	113,500 (34.4)	95,820 (29.3)^d^	309.4 (30.8)	1.52 (0.52–3.00)	40.20 (51.1)^c^	19.6 (21.9)	0.212 (34.4)	5.97 (31.9)	5.77 (29.8)

*AUC* area under the serum dostarlimab concentration–time curve, *DLT-eval* dose-limiting toxicity-evaluable, *GCV%* geometric coefficient of variation, *ND* not determined, *PK/PDy* pharmacokinetics/pharmacodynamics, *V*_*z*_ volume of distribution during terminal elimination

Note that AUC_(0-τ)_ is calculated based on the 28 days for PK/PDy patients and 14 days for all other patients

^a^Median (range)

^b^*C*_trough_ at nominal time of 336 h for DLT-evaluable patients and 504 h for PK/PDy patients

^c^*n* = 2; only minimum and maximum presented

^d^*n* = 5

^e^AUC_(0-*τ*)_ is identical to AUC_(0-last)_, and hence only one parameter is presented

^f^*C*_trough_ at nominal of 504 h for patients with Q3W dosing and 1008 h for patients with Q6W dosing

Dose proportionality was evaluated for doses ranging from 1 to 10 mg/kg (part 1) based on the single-dose data from cycle 1. PK/PDy patients were excluded from analysis. For the first dose of the DLT cohorts (*n* = 3, 3, and 6 for 1, 3, and 10 mg/kg doses, respectively), the *C*_max_ was 21.78, 66.17, and 228.4 µg/mL at the 1, 3, and 10 mg/kg doses, respectively. The respective AUC_0-tau_ was 3417, 10,790, and 36,480 µg h/mL. The respective AUC_0-inf_ was 4440 to 9570, 26,630, and 59,830 µg h/mL. Statistical assessment of dostarlimab dose proportionality for part 1 over a range of 1–10 mg/kg in cycle 1 is presented in Table 2. These results demonstrate that the 90% confidence interval (CI) for the slope encompasses one for all three parameters. Therefore, dostarlimab displays dose-proportional PK over the dose range of 1–10 mg/kg.

**Table 2:** Summary of assessment for dostarlimab dose proportionality, part 1 cycle 1

Pharmacokinetic parameter	*n*	Slope		
		Estimate	Standard error	90% CI
*C*_max_ (µg/mL)	12	1.02	0.064	(0.91–1.13)
AUC_(0-∞)_ (µg**·**h/mL)	11	0.91	0.11	(0.71–1.10)
AUC_(0-τ)_ (µg**·**h/mL)	12	1.03	0.07	(0.90–1.15)

*AUC* area under the serum dostarlimab concentration–time curve, *C*_*max*_ maximum serum dostarlimab concentration

During cycle 1, median *t*_max_ values were observed shortly after the end of the scheduled 0.5-h infusion and ranged from 1.50 to 2.92 h in part 1 and 0.96 to 1.52 h in part 2A.

There were no clear trends for dose-dependent changes in clearance, steady-state volume of distribution (*V*_ss_), or terminal elimination half life (*t*_1/2_) in cycle 1 of part 1, but it should be noted that the sample size was small (Table 1). For part 2A, geometric mean values were within the range observed for part 1 data (14.5 and 19.6 days for *t*_1/2_, 0.216 and 0.212 L/day for clearance, and 4.38 and 5.77 L for *V*_ss_ for 500 mg Q3W and 1000 mg Q6W, respectively).

Following multiple dose administration in parts 1 and 2A, observed accumulation ranged from 180 to 374% for AUC_(0-*τ*)_ and 126 to 259% for *C*_max_, which is generally consistent with dosing regimen and observed *t*_1/2_. Although based on limited available data, multiple-dose cycles generally indicated an intraindividual decrease in clearance compared with cycle 1.

**Fig. 1:**
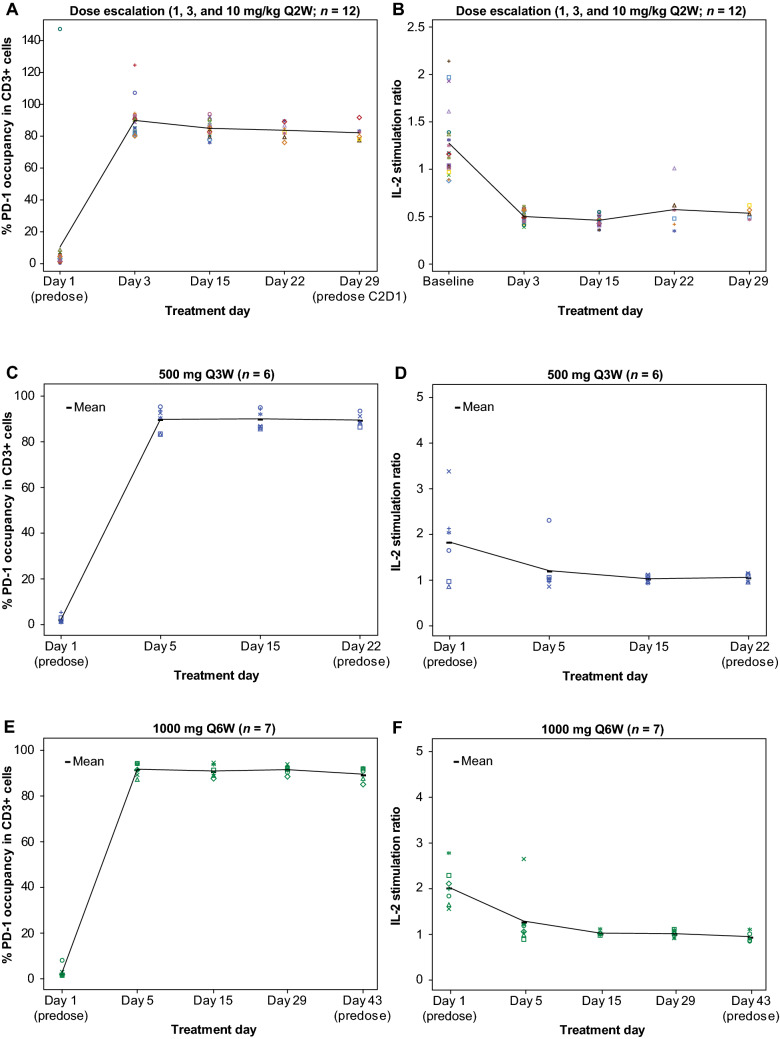
*CD3+ binding assays and interleukin-2 (IL-2) stimulation assays demonstrate PD-1 receptor occupancy by dostarlimab following administration of dostarlimab 1, 3, and 10 mg/kg (CD3+ : A; IL-2: B) and dostarlimab 500 mg Q3W (CD3+ : C; IL-2: D) and 1000 mg Q6W (CD3+ : E; IL-2: F). *Anomalous data were observed infrequently within the data sets; however, no obvious issue in sample handling or processing could be identified

In parts 1 and 2A, maximally achievable RO was observed based on the both CD3+ binding and IL-2 stimulation assays throughout each treatment cycle and across all dosage regimens evaluated (Fig. 1A–F). The mean minimum concentration at which full RO was observed was 2.44 µg/mL. Only 1 PK/PDy patient did not achieve full RO: patient (94 kg) received dostarlimab 1 mg/kg on day 1 of cycle 1; full RO was not reached by day 22 sample at a drug concentration of 1.51 µg/mL. This patient tested positive for anti-dostarlimab antibodies and was included in all analyses.

A two-compartment model best described observed PK data in part 1 and was used to predict the dose and regimen. Dose selection for part 2A was guided primarily by the observed RO data from peripheral blood cells, in addition to safety and PK data from patients in part 1. Full RO was achieved at serum concentrations of 2.44 μg/mL and above. The model predicted *C*_trough_ at steady state for the 500 mg Q3W and 1000 mg Q6W of 51.1 (90% CI, 13.4–111.1 μg/mL) and 29.2 μg/mL (90% CI, 4.1–78.5 μg/mL), respectively. Importantly, these doses are projected to result in 90% lower bound of the mean predicted *C*_trough_ following 500 mg Q3W and 1000 mg Q6W dosing that is 5.5-fold and 1.7-fold, respectively, of the level required for full RO of peripheral blood cells.

**Fig. 2:**
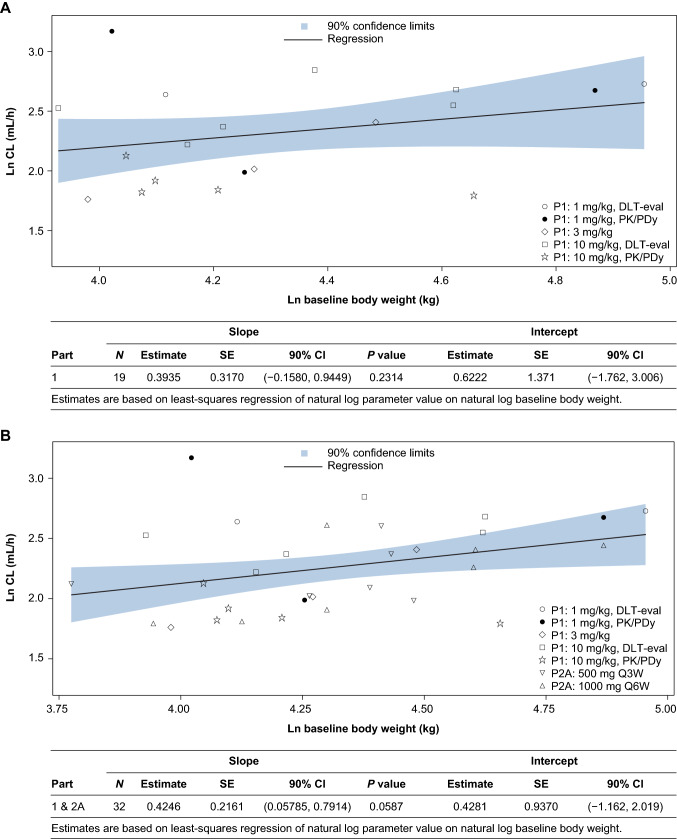
Individual dostarlimab clearance versus body weight on day 1 of cycle 1 for part 1 (**A**), part 1 plus 2A (**B**), and intercept and slope data for the populations in **A** and **B**

Body weight within the range of 45.6–145.6 kg was evaluated as a covariate for clearance. Although there was a trend toward slightly higher clearance with higher body weight, it was not statistically significant (Fig. 2A). Based on these results, 2 fixed-dose regimens of dostarlimab (500 mg Q3W and 1000 mg Q6W) were explored in part 2A to gain additional confidence around PK and safety of these dosing schedules. Both dose regimens were found to be safe without any DLTs and revealed dose-proportional PK. As shown in Table 1, values for *C*_trough_ and *τ*, normalized AUC after administration of either fixed-dose regimen, were comparable. There was at least an 8.5-fold margin between serum dostarlimab concentrations achieved during the whole treatment process in part 2A and the lowest serum dostarlimab concentration required for full RO. This margin is supported after consideration of exposure interpatient variability and a typical threefold tissue dilution expected of mAbs. Hence, these doses and associated plasma exposures will allow for delivery of dostarlimab concentrations to tumor sites that are sufficient for potential antitumor activity [20].

Combining data from parts 1 and 2A (Fig. 2B) confirmed the trend of increasing clearance with increasing baseline body weight. However, this relationship was not statistically significant at the 0.05 level.

### Recommended therapeutic (fixed) dose

No DLTs were observed in part 1 (weight-based doses Q2W) or part 2A (500 mg Q3W and 1000 mg Q6W). The MTD was not reached. The RTD of dostarlimab was determined as 500 mg Q3W for the first four cycles, followed by 1000 mg Q6W. This dose regimen was developed for clinical convenience. Early Q3W dosing facilitates monitoring of patients receiving a new agent, then simplifies to less frequent Q6W dosing after the initial monitoring period.

### Safety

All patients in parts 1 and 2A experienced at least 1 treatment-emergent AE (Table 3). Of 34 patients in parts 1 and 2A, 12 (35.3%) had grade 3 or higher AEs; 4 (11.8%) were considered treatment-related: part 1, aspartate transaminase and alanine transaminase elevations (dostarlimab 10 mg/kg) and increased lipase (dostarlimab 1 mg/kg); and part 2A, fatigue (dostarlimab 500 mg Q3W) and pneumonitis (dostarlimab 1000 mg Q6W) (Table 3). The grade 3 aspartate and alanine transaminase elevations in one patient receiving dostarlimab 10 mg/kg in part 1 led to drug withdrawal. These AEs resolved with corticosteroid therapy. Two immune-mediated AEs were observed: arthritis (grade 2, part 1) and pneumonitis (grade 3, part 2A, same event listed among treatment-related grade ≥ 3 AEs above).

**Table 3:** Treatment-emergent adverse events (AEs)

Summary, *n* (%)	Part 1 dostarlimab dose levels				Part 2A dostarlimab regimens		
	1 mg/kg, *n* = 6	3 mg/kg, *n* = 3	10 mg/kg, *n* = 12	Total, *N* = 21	500 mg Q3W, *n* = 6	1000 mg Q6W, *n* = 7	Total *N*, = 13
Any AE	6 (100)	3 (100)	12 (100)	21 (100)	6 (100)	7 (100)	13 (100)
Dostarlimab-related AE	6 (100.0)	2 (66.7)	9 (75.0)	17 (81.0)	5 (83.3)	5 (71.4)	10 (76.9)
Any grade ≥ 3 AE	3 (50.0)	1 (33.3)	6 (50.0)	10 (47.6)	1 (16.7)	1 (14.3)	2 (15.4)
Dostarlimab-related grade ≥ 3 AE	1 (16.7)	0	1 (8.3)	2 (9.5)	1 (16.7)	1 (14.3)	2 (15.4)
Any serious AE	2 (33.3)	0	6 (50.0)	8 (38.1)	1 (16.7)	1 (14.3)	2 (15.4)
Dostarlimab-related serious AE	0	0	1 (8.3)^a^	1 (4.8)	0	1 (14.3)^b^	1 (7.7)
Any AE leading to drug withdrawal	0	0	2 (16.7)	2 (9.5)	0	1 (14.3)	1 (7.7)
Dostarlimab-related AE leading to drug withdrawal	0	0	1 (8.3)	1 (4.8)	0	1 (14.3)	1 (7.7)
Any AE leading to treatment interruption	1 (16.7)	2 (66.7)	3 (25.0)	6 (28.6)	2 (33.3)	0	2 (15.4)
Any immune-related AE	0	0	1 (8.3)	1 (4.8)	0	1 (14.3)	1 (7.7)
Type of treatment-related AEs^c^							
Fatigue	1 (16.7)	2 (66.7)	4 (33.3)	7 (33.3)	2 (33.3)	3 (42.9)	5 (38.5)
Nausea	1 (16.7)	1 (33.3)	3 (25.0)	5 (23.8)	1 (16.7)	0	1 (7.7)
Pruritus	2 (33.3)	1 (33.3)	1 (8.3)	4 (19.0)	0	0	0
Arthralgia	1 (16.7)	1 (33.3)	1 (8.3)	3 (14.3)	1 (16.7)	0	1 (7.7)
Decreased appetite	2 (33.3)	1 (33.3)	0	3 (14.3)	1 (16.7)	0	1 (7.7)
Maculopapular rash	2 (33.3)	1 (33.3)	0	3 (14.3)	0	0	0
Alopecia	0	1 (33.3)	1 (8.3)	2 (9.5)	0	0	0
Vomiting	0	0	2 (16.7)	2 (9.5)	0	0	0
Anemia	0	0	0	0	1 (16.7)	0	1 (7.7)
Amylase increased	0	0	0	0	1 (16.7)	1 (14.3)	2 (15.4)
Hypokalemia	1 (16.7)	0	0	1 (4.8)	1 (16.7)	1 (14.3)	2 (15.4)

^a^Alanine aminotransferase and aspartate aminotransferase increased

^b^Pneumonitis

^c^Incidence ≥ 10% in each study part by Medical Dictionary for Regulatory Activities preferred term

### Preliminary antitumor activity

In part 1, all 21 patients had a follow-up scan and were evaluable for response (Supplementary Figure S3). Two (9.5%) patients had irPRs as best response. One responder had ovarian cancer (OC) and was treated at the 3 mg/kg dose level (time to response, 3 months; duration of response, 4.9 months). The other responder had small cell lung cancer and was treated at the 10 mg/kg dose level (time to response, 4.5 months; duration of response, 13.6 months). Five (23.8%) patients had stable disease (1 mg/kg, *n* = 2; 3 mg/kg, *n* = 1; 10 mg/kg, *n* = 2). Immune-related overall response rate was 9.5%, and disease control rate was 33.3%. One patient with OC had been receiving ongoing treatment with dostarlimab for more than 2 years with stable disease.

In part 2A, all 13 patients had follow-up scanning and were evaluable for response. Two patients who received dostarlimab 1000 mg Q6W achieved stable disease.

## Discussion

Dostarlimab PK were dose proportional, and full RO was observed at all dose levels throughout the dosing cycle in weight-based and fixed-dose cohorts using assays similar to those used for nivolumab [14] and pembrolizumab [15]. Dostarlimab *C*_trough_ after administration of dostarlimab 500 mg Q3W (geometric mean of 39.17 µg/mL) was similar to that after dostarlimab 1000 mg Q6W (geometric mean of 40.20 µg/mL) and well above the lowest dostarlimab concentration needed for full peripheral RO even after accounting for interpatient variability and typical tissue dilution with mAbs. Coefficients of variability for exposure were not better with weight-based dosing than with fixed-dose schedules. No DLTs were observed in this study. Dostarlimab demonstrated encouraging clinical activity in heavily pretreated patients with diverse tumor types, which was comparable to another PD-1 inhibitor (pembrolizumab) in this setting [15].

Maximally achievable direct and functional ROs were observed with CD3+ binding and IL-2 stimulation assays throughout each treatment cycle with all dostarlimab dosage regimens evaluated. As our population PK model included 1 patient who failed to attain full RO at 1 mg/kg by day 22 and had anti-dostarlimab antibodies, it is possible that the *C*_trough_ margin provided by the RTD relative to the concentration required for full RO will be sufficient for patients with antidrug antibodies, who may potentially gain efficacy from dostarlimab. In studies of other anti-PD-1 IgG4 mAbs, direct RO was saturated at a nivolumab dosage of at least 0.3 mg/kg [21], and 95% target engagement based on the IL-2 stimulation assay was achieved by pembrolizumab at a dosage of 2 mg/kg Q2W [22].

The antitumor activity in the portion of the study reported here (parts 1 and 2A) was a secondary endpoint and was signal seeking in nature. Subsequent patients have enrolled into disease-specific cohorts in part 2B, where the primary objective response is antitumor activity by objective response rate and duration of response. At an interim analysis with the data cut in March 2020, patients with advanced or recurrent dMMR endometrial cancer had an observed ORR of 44.7% (*N* = 103) [23], and in patients with dMMR nonendometrial advanced solid tumors, an ORR of 38.7% was observed (*N* = 106) [24].

Preliminary safety findings demonstrated that dostarlimab was well-tolerated, with no DLTs across the doses tested. Most treatment-related AEs were low grade and manageable; dostarlimab exhibited an expected safety profile for a PD-1 inhibitor [7, 8]. Immune-mediated AEs associated with dostarlimab were infrequent.

### Limitations

A number of anti-PD-(L)1 mAbs are approved already, and much is known on the characteristics of these mAbs using their established dosing regimens. Pembrolizumab was approved with a Q6W dosing regimen based on the population modeling data. Dostarlimab is the first anti-PD-1 mAb with prospective preapproval clinical patient data using a Q6W dosing regimen. Although safety and clinical activity were seen in this patient sample at Q2W, Q3W, and Q6W dosing, further study in more patients will be required to better understand the full implications (including safety, efficacy, and patient-reported outcomes) of this unique variable-dosing regimen.

## Conclusion

Overall, this first-in-human study of dostarlimab monotherapy showed favorable PK, proof-of-concept PD, safety, and tolerability, and encouraging antitumor activity in patients with advanced tumors. Based on the safety, PK, and RO data, the dostarlimab RTD of 500 mg Q3W for the first 4 cycles followed by 1000 mg Q6W thereafter is undergoing further clinical development in patients with multiple tumor types.

## Supplementary Information

Below is the link to the electronic supplementary material.

Supplementary file1 (DOCX 557 KB)

## Data Availability

Anonymized individual participant data and study documents can be requested for further research from www.clinicalstudydatarequest.com
