# Supplementary material for: Safety, antitumor activity, and pharmacokinetics of dostarlimab, an anti-PD-1, in patients with advanced solid tumors: a dose–escalation phase 1 trial
Source: Cancer Chemother Pharmacol. 2021 Nov 8;89(1):93–103. doi: 10.1007/s00280-021-04358-3 (PMC8739161; doi:10.1007/s00280-021-04358-3)
Supplement: Supplementary file 1 — Supplementary file1 (DOCX 557 KB) [file 280_2021_4358_MOESM1_ESM.docx]

**ADDITIONAL FILES:**

**Safety, Antitumor Activity, and Pharmacokinetics of Dostarlimab, an Anti–PD-1 in Patients With Advanced Solid Tumors: A Dose Escalation Phase 1 Trial**

A. Patnaik, G.J. Weiss, D.W. Rasco, L. Blaydorn, A. Mirabella, M. Beeram, W. Guo, S. Lu, H. Danaee, K. McEachern, E. Im, J.C. Sachdev

**Supplementary Methods**

PK sample collection

In part 1, blood samples for assessment of dostarlimab PK were collected predosing and at 0.25, 0.5, 1.5, 3, 24, 48, 96, 168, 336 (day 15), and 504 (day 22, PK/PDy patients only) hours after the start of infusion on cycle 1 day 1. For DLT-evaluable patients, 2 doses were received in cycle 1: 1 on day 1 and 1 on day 15. For these patients, blood taken at 336 hours was the last sampling point for cycle 1 day 1. During cycles 2 to 6, blood samples were collected predosing and at 0.25 and 0.5 hours after infusion start on days 1 and 15, as well as at 1.5, 3, 24, 96, 168, and 336 (day 15) hours after infusion start on cycle 6 day 1. A predosing sample was obtained on day 1 of cycle 8 and every other cycle thereafter. A similar sampling scheme was deployed for part 2A, blood samples for assessment of dostarlimab PK in the every 3 weeks (Q3W) treatment arm were collected predosing and at 0.25, 0.5, 1.5, 3, 24, 48, 96, 168, and 336 hours following the start of infusion on day 1 of cycles 1 and 5. In cycles 2, 3, and 4, blood samples were collected predosing and then 0.25 and 0.5 hours postdosing. A predosing sample was obtained on day 1 of cycle 6 and of every 3 cycles thereafter. In the Q6W treatment arm, blood samples for assessment of dostarlimab PK were collected predosing and at 0.25, 0.5, 1.5, 3, 24, 48, 96, 168 (day 8), 336 (day 15), 504 (day 21), 672 (day 28), and 840 (day 35) hours following the start of infusion on day 1 of cycles 1 and 4. For cycles 2 and 3, blood samples were collected predosing and then 0.25 and 0.5 hours postdosing. A predosing sample was obtained on day 1 of cycle 5 and of every 3 cycles thereafter.

Dostarlimab assay

**Dostarlimab assay**

Concentrations of dostarlimab in serum at each time point were analyzed by enzyme-linked immunosorbent assay (ELISA). The 4 key reagents used for the sample analysis were dostarlimab (lot APE07042.01) at 20.7 mg/mL, capturing antigen recombinant human PD-1 mouse IgG2a Fc fusion protein (lot APE01696.09; AnaptysBio Inc., San Diego, CA) at 2.16 mg/mL, detection antibody biotinylated anti–human IgG4 (Abcam #ab99818, Cambridge, MA) at 0.5 mg/mL, and working conjugate Streptavidin-HRP conjugate (Sigma #GERPN1231-2mL).

The plate was coated with recombinant human PD-1 mouse IgG2a Fc fusion protein and blocked by 1% bovine serum albumin (BSA). Dostarlimab standards, quality controls, and samples with the minimum required dilution of 1:10 in human serum were added to wells and incubated for approximately 1 hour at 25 ± 2ºC on a Jitterbug shaker at 575 rpm. Detection antibody of 1:18,000 dilution (in 0.1% BSA in 1x phosphate-buffered saline [PBS]) and Streptavidin-HRP conjugate of 1:18,000 dilution (in 0.1% BSA in 1x PBS) were sequentially added and incubated for 1 hour. Streptavidin-HRP, which binds to biotinylated anti-human IgG4, reacts with tetramethylbenzidine (TMB), changes the color of TMB with the stop solution (KPL #50-85-06), and generates an absorbance signal at 450 nM (SpectraMax M5 SoftMax Pro v.6.4, Molecular Devices LLC, San Jose, CA). The absorbance was proportional to the amount of dostarlimab in the standards, quality controls, and samples.

The signals from standards were fitted with a 5-parameter curve using 1/Y weighting. The quality controls, blanks, and samples were quantified against the curve. The assay was validated for the range of 32 to 814 ng/mL (lower limit of quantification [LLOQ], 32.0 ng/mL), with intrarun accuracy (% bias) and precision (% coefficient of variation [CV]) ranging from −21.6% (LLOQ) to 11.4% and 0.5% to 9.6%, respectively; interrun accuracy (% bias), precision, and percentage of total error (including failed batches) ranged from −6.3% to 3.5%, 5.1% to 12.9%, and 7.7% to 19.1%, respectively. There was no hook effect observed with dilution linearity from 700 µg/mL. Nine out of 10 lots of cancer serum passed the selectivity criteria. The stabilities were 24 hours at room temperature and refrigerated (2 ºC to 8 ºC), and 6 freeze/thaw cycles (−80 ºC/room temperature). There was no impact for accuracy and precision in either hemolyzed or lipemic serum. The assay was evaluated for a range of incubating time for each step of the assay and proved to be robust.

**Supplementary Table S1** Guidelines for dose delay or modification of dostarlimab

| **Event(s)** | **Dose delay or modification** |
| --- | --- |
| General | - Part 1: Intrapatient dose escalation to a dose that has been tested and shown to be safe during dose escalation may be permitted following an agreement between the investigator and sponsor - Part 2A: If Q3W and Q6W doses are declared safe, patients enrolled in the Q3W cohort are permitted to change to Q6W dose schedule after having received at least 4 cycles of dostarlimab doses |
| Medical/surgical events or logistical reasons not related to study therapy (eg, surgery, unrelated medical events, patient vacation, and/or holidays) | - Study treatment dosing delays were permitted. Patients were placed back on study therapy within 28 days of the scheduled dostarlimab infusion. If a delay was >28 days, the patient was placed back on study therapy only after discussion with the sponsor. Reasons for treatment delays of >3 days were documented. |
| Adverse events (AEs) | - AEs (nonserious and serious) associated with dostarlimab exposure may have represented an immunologic etiology. These AEs may occur shortly after the first dose or several months after the last dose of treatment. - In general, dostarlimab must have been withheld for drug-related grade 3 toxicities but resumed on recovery to grade ≤1; dostarlimab was permanently discontinued for any drug-related grade 4 event. Dostarlimab was discontinued for some grade 3 immunologic-mediated AEs as described in Additional file 3: Supplementary Table S2. |

*AE* adverse event, *QXW* every X weeks

**Supplementary Table S2** Guidelines for treatment of immune-related adverse events (AEs) of interest

| **Toxicity** | **Hold treatment for grade** | **Restarting treatment/discontinuation** |
| --- | --- | --- |
| Diarrhea/colitis | 2–3 | Restart dosing when toxicity resolves to grade 0–1 |
|  | 4 | Permanently discontinue |
| AST, ALT, or increased bilirubin | 2 (AST or ALT >3 and ≤5 × ULN or  total bilirubin >1.5 and ≤3 × ULN) | Restart dosing when toxicity resolves to grade 0–1 |
|  | 3–4 (AST or ALT >5 × ULN  or total bilirubin >3 × ULN) | Permanently discontinue (see exception below)^a^ |
| T1DM or hyperglycemia | 3–4 hyperglycemia or T1DM (associated with metabolic acidosis or ketonuria) | Restart dosing in appropriately managed, clinically and metabolically stable patients; insulin replacement therapy is required |
| Immune-related encephalitis | Any grade | Permanently discontinue |
| Hypophysitis | 2–4 | For grade 2–3, hold until hormonal therapy results in return to adequate levels by laboratory values and restart dosing when toxicity resolves to grade 0–1. For recurrence or worsening of grade ≥2 hypophysitis after steroid taper has been completed and patient is on adequate hormone replacement therapy, permanently discontinue. For grade 4, permanently discontinue |
| Adrenal insufficiency | 2–3 | Hold until hormonal therapy results in return to adequate levels by laboratory values and restart dosing when toxicity resolves to grade 0–1. For recurrent or worsening grade ≥2 adrenal insufficiency while an adequate hormonal replacement is continuing, permanently discontinue study drug |
|  | 4 | Permanently discontinue |
| Hypothyroidism or hyperthyroidism | 3 | Hold until hormonal therapy results in return to adequate levels by laboratory values and restart dosing when toxicity resolves to grade 0–1 |
|  | 4 | Permanently discontinue |
| Infusion-related reaction | 2^b^ | Restart dosing when toxicity resolves to grade 0–1 |
|  | 3–4 | Permanently discontinue |
| Pneumonitis | 2 | Restart dosing when toxicity resolves to grade 0–1. If grade 2 recurs, permanently discontinue |
|  | 3–4 | Permanently discontinue |
| Rash | 3 | Restart dosing when toxicity resolves to grade 0–1 |
|  | 4 | Permanently discontinue |
| Renal failure or nephritis | 2 | Restart dosing when toxicity resolves to grade 0–1 |
|  | 3–4 | Permanently discontinue |
| Recurrence of AEs after resolution to grade ≤1 | 3–4 | Permanently discontinue |

*ALT* alanine aminotransferase, *AST* aspartate aminotransferase, *T1DM* type 1 diabetes mellitus, *ULN* upper limit of normal

^a^For patients with liver metastasis who begin treatment with grade 2 AST or ALT, if AST or ALT increases by ≥50% relative to baseline and lasts for at least 1 week, then the treatment should be discontinued

^b^Upon resolution within 1 hour of stopping drug infusion, the infusion may be restarted at 50% of the original infusion rate (eg, from 100 mL/h to 50 mL/h). Otherwise, dosing will be held until symptoms resolve, and the patient should be premedicated for the next scheduled dose

**Supplementary Table S3** Patient eligibility criteria

| **Inclusion criteria** |
| --- |
| At least 18 years of age with any histologically or cytologically proven recurrent or advanced solid tumor, and with disease progression after treatment with available anticancer therapies, or intolerance to such treatment. Female patients had a negative serum pregnancy test within 72 hours of the first dose of study medication, unless they were of nonchildbearing potential. Female patients of childbearing potential agreed to use 2 adequate methods of contraception with their partners, starting with the screening visit through 150 days after the last dose of study therapy. Patients had an Eastern Cooperative Oncology Group performance status of ≤2 for part 1 and ≤1 for part 2. Patients had adequate organ function, defined as absolute neutrophil count ≥1500/µL; platelets ≥100,000/µL; hemoglobin ≥9 g/dL or ≥5.6 mmol/L; serum creatinine ≤1.5 × upper limit of normal (ULN) or calculated creatinine clearance ≥50 mL/min using Cockcroft-Gault equation for patients with creatinine levels >1.5 × institutional ULN); total bilirubin ≤1.5 × ULN *and* direct bilirubin ≤1 × ULN; aspartate aminotransferase and alanine aminotransferase ≤2.5 × ULN unless liver metastases were present, in which case they must have been ≤5 × ULN; international normalized ratio or prothrombin time (PT) ≤1.5 × ULN unless patient was receiving anticoagulant therapy as long as PT or PTT (partial thromboplastin time) was within therapeutic range of intended use of anticoagulants; and activated PTT ≤1.5 × ULN unless patient was receiving anticoagulant therapy, as long as PT or PTT was within therapeutic range of intended use of anticoagulants. |
| **Exclusion criteria** |
| Prior therapy with an anti–PD-1, anti–PD-L1, or anti–PD-L2 agent; known uncontrolled central nervous system metastases and/or carcinomatous meningitis, or additional malignancy that progressed or required active treatment within the last 2 years; known history of human immunodeficiency virus, hepatitis B or C, or interstitial lung disease; or active autoimmune disease that required systemic treatment in the last 2 years. |

*PD-1* programmed death receptor 1, *PD-L1/2* PD 1/2 ligand

**Supplementary Table S4** Dose-limiting toxicity criteria as assessed during cycle 1 in part 1 and cycle 1 in part 2A

| - In part 1, the MTD was defined as 1 dose level below the dose level deemed unsafe based on a modified 3 + 3 design. In part 2A, a modified 6 + 6 design was used to evaluate dostarlimab safety at the fixed-dose levels of 500 mg Q3W and 1000 mg Q6W. |
| --- |
| - Any treatment-related grade ≥3 nonhematologic clinical (nonlaboratory) toxicity, excluding:   - Nausea and vomiting resolving to grade ≤1 within 48 hours   - Grade 3 diarrhea with duration <48 hours   - Grade 3 fatigue with duration <7 days   - Infusion-related reaction grade ≥3 or recurrent infusion-related reaction ≥2 despite adequate premedication |
| - Any treatment-related nonhematologic toxicity specifically defined as:   - Grade ≥2 uveitis, eye pain, or blurred vision that does not resolve with topical therapy within 2 weeks   - Grade ≥2 immune-related endocrine toxicity that requires hormone replacement (except grade 2 thyroiditis or thyroid dysfunction)   - Grade ≥2 colitis or diarrhea that persists for ≥7 days despite adequate steroid therapy   - Any toxicity that results in a treatment delay of ≥7 |
| - Any treatment-related grade ≥3 nonhematologic laboratory abnormality if:   - Medical intervention is required to treat the patient, or   - The abnormality leads to hospitalization, or   - The abnormality persists for ≥7 days |
| - Any treatment-related hematologic toxicity specifically defined as:   - Grade 4 thrombocytopenia for ≥7 days, or grade 3 or 4 thrombocytopenia associated with bleeding or requiring platelet transfusion   - Grade 4 neutropenia for ≥7 days, or grade 3 or 4 neutropenia associated with infection or febrile neutropenia   - Grade 4 anemia, or grade 3 anemia requiring a blood transfusion |

*MTD* maximum tolerated dose, *QXW* every X weeks

**Supplementary Table S5** Patient demographics and clinical characteristics at baseline

| **Parameter** | **Part 1 dostarlimab dose levels (all doses were Q2W in** p**art 1)** | | | | | **Part 2A dostarlimab regimens** | | |
| --- | --- | --- | --- | --- | --- | --- | --- | --- |
|  | 1 mg/kg, *n* = 6 | 3 mg/kg, *n* = 3 | 10 mg/kg, *n* = 12 | | Total, *N* = 21 | 500 mg Q3W, *n* = 6 | 1000 mg Q6W, *n* = 7 | Total, *N* = 13 |
| Age, median (IQR), y | 55.5  (45.0–61.0) | 72.0  (56.0–86.0) | 56.5  (47.5–63.5) | | 57.0  (49.0–66.0) | 70.5  (61.0–77.0) | 57.0  (49.0–71.0) | 67.0  (53.0–74.0) |
| Female, *n* (%) | 5 (83.3) | 2 (66.7) | 10 (83.3) | | 17 (81.0) | 6 (100.0) | 4 (57.1) | 10 (76.9) |
| ECOG PS, *n* (%) |  |  |  | |  |  |  |  |
| 0 | 3 (50.0) | 1 (33.3) | 4 (33.3) | | 8 (38.1) | 2 (33.3) | 2 (28.6) | 4 (30.8) |
| 1 | 3 (50.0) | 2 (66.7) | 8 (66.7) | | 13 (61.9) | 4 (66.7) | 5 (71.4) | 9 (69.2) |
| Tumor site, *n* (%) |  |  |  | |  |  |  |  |
| Ovarian^a^ | 2 (33.3) | 2 (66.7) | 4 (33.3) | | 7 (33.3) | 1 (16.7) | 3 (42.9) | 2 (15.4) |
| Breast | 2 (33.3) | 0 | 2 (16.7) | | 3 (14.3) | 0 | 0 | 0 |
| Colon | 0 | 0 | 1 (8.3) | | 1 (4.8) | 0 | 0 | 0 |
| Endometrial | 0 | 0 | 0 | | 0 | 1 (16.7) | 0 | 1 (7.7) |
| Esophageal | 0 | 0 | 1 (8.3) | | 1 (4.8) | 1 (16.7) | 0 | 0 |
| Lung | 0 | 0 | 1 (8.3) | | 1 (4.8) | 0 | 0 | 0 |
| Pancreas | 0 | 0 | 1 (8.3) | | 1 (4.8) | 1 (16.7) | 0 | 1 (7.7) |
| Peritoneum | 0 | 0 | 1 (8.3) | | 1 (4.8) | 0 | 0 | 0 |
| Prostate | 0 | 1 (33.3) | 0 | | 1 (4.8) | 0 | 0 | 0 |
| Kidney | 0 | 0 | 0 | | 0 | 0 | 1 (14.3) | 1 (7.7) |
| Rectum | 0 | 0 | 0 | | 0 | 0 | 1 (14.3) | 1 (7.7) |
| Other | 2 (33.3) | 0 | 1 (8.3) | | 5 (23.8) | 2 (33.3) | 1 (14.3) | 6 (46.2) |
| No. of prior regimens, median (IQR) | 7.0 (1–10) | 2.0 (2–4) | 4.0 (2–7) | | 4.0 (2–7) | 4.0 (2–8) | 2.0 (1–4) | 3.0 (1–4) |
| No. of prior regimens for metastatic disease, median (IQR) | 6.0 (0–10) | 2.0 (2–3) | 3.5 (2–7) | | 3.0 (2–7) | 1.0 (0–6) | 1.0 (1–1) | 1.0 (0–1) |
| Type of previous treatment, *n* (%) | | | | | | | | |
| Bevacizumab | 1 (16.7) | 0 | | 4 (33.3) | 5 (23.8) | 1 (16.7) | 4 (57.1) | 5 (38.5) |
| Capecitabine | 2 (33.3) | 0 | | 2 (16.7) | 4 (19.0) | 0 | 2 (28.6) | 2 (15.4) |
| Carboplatin | 2 (33.3) | 1 (33.3) | | 5 (41.7) | 8 (38.1) | 1 (16.7) | 1 (14.3) | 2 (15.4) |
| Carboplatin + gemcitabine | 0 | 0 | | 1 (8.3) | 1 (4.8) | 1 (16.7) | 0 | 1 (7.7) |
| Cisplatin | 1 (16.7) | 0 | | 5 (41.7) | 6 (28.6) | 2 (33.3) | 2 (28.6) | 4 (30.8) |
| Cyclophosphamide | 1 (16.7) | 0 | | 1 (8.3) | 2 (9.5) | 1 (16.7) | 0 | 1 (7.7) |
| Cyclophosphamide + doxorubicin | 0 | 0 | | 1 (8.3) | 1 (4.8) | 0 | 0 | 0 |
| Cyclophosphamide + doxorubicin + fluorouracil | 1 (16.7) | 0 | | 0 | 1 (4.8) | 0 | 0 | 0 |
| Docetaxel | 1 (16.7) | 1 (33.3) | | 3 (25.0) | 5 (23.8) | 0 | 0 | 0 |
| Doxorubicin | 3 (50.0) | 0 | | 4 (33.3) | 7 (33.3) | 0 | 1 (14.3) | 1 (7.7) |
| Etoposide | 0 | 0 | | 1 (8.3) | 1 (4.8) | 0 | 0 | 0 |
| Fluorouracil | 2 (33.3) | 0 | | 1 (8.3) | 3 (14.3) | 0 | 1 (14.3) | 1 (7.7) |
| Gemcitabine | 3 (50.0) | 0 | | 4 (33.3) | 7 (33.3) | 0 | 1 (14.3) | 1 (7.7) |
| Irinotecan | 0 | 0 | | 1 (8.3) | 1 (4.8) | 0 | 1 (14.3) | 1 (7.7) |
| Methotrexate | 1 (16.7) | 0 | | 0 | 1 (4.8) | 0 | 0 | 0 |
| NAB-paclitaxel | 1 (16.7) | 0 | | 0 | 1 (4.8) | 0 | 0 | 0 |
| *ECOG* Eastern Cooperative Oncology Group, *IQR* interquartile range, *NAB* nanoparticle albumin bound, *PS* performance status, *QXW* every X weeks  ^a^Includes patients with ovarian or fallopian tube as the site of tumor origin | | | | | | | | |

**Supplementary Table S6** Summary statistics for dostarlimab pharmacokinetic parameters, part 1 cycle 6 and part 2a cycle 5 (Q3W) and cycle 4 (Q6W)

|  | **Part 1 cycle 6: geometric mean (GCV%)** | | | | | | | | | |
| --- | --- | --- | --- | --- | --- | --- | --- | --- | --- | --- |
|  | **AUC_(0–τ)_ (µg·h/mL)** | ***C*_max_  (µg/mL)** | ***t*_max_^a^ (h)** | ***C*_min_  (µg/mL)** | ***t*_min_^a^ (h)** | ***t*_1/2_ (h)** | ***CL*  (mL/h)** | ***V*_ss_   (mL)** | **RAUC_(0–τ)_ (%)** | **RC_max_ (%)** |
| 1 mg/kg, DLT-eval  (*n* = 1) | 8970 | 42.1 | 1.43 | 17.3 | 336.23 | 292 | 14.9 | 6250 | 184 | 145 |
| 1 mg/kg, PK/PDy  (*n* = 1) | ND (n = 0) | ND (n = 0) | ND (n = 0) | ND (n = 0) | ND (n = 0) | ND (n = 0) | ND (n = 0) | ND (n = 0) | ND (n = 0) | ND (n = 0) |
| 3 mg/kg  (*n* = 2) | 31,800–43,500 | 156–186 | 2.27 (1.52–3.02) | 71.2–110 | 167.19 (0.00–334.38) | 423^b^ | 3.68–6.67 | 4130^b^ | 328–374 | 230–259 |
| 10 mg/kg, DLT-eval  (*n* = 1) | 96,000 | 395 | 1.55 | 216 | 0.00 | 595 | 6.25 | 5400 | 302 | 223 |
| 10 mg/kg, PK/PDy  (*n* = 1) | 121,000 | 531 | 1.52 | 214 | 0.00 | 571 | 8.71 | 7000 | 197 | 152 |
|  | **Part 2A cycle 5 (Q3W) and cycle 4 (Q6W): geometric mean (GCV%)** | | | | | | | | | |
|  | **AUC_(0–τ)_ (µg·h/mL)** | ***C*_max_  (µg/mL)** | ***t*_max_^a^ (h)** | ***C*_min_  (µg/mL)** | ***t*_min_^a^ (h)** | ***t*_1/2_ (h)** | ***CL*  (mL/h)** | ***V*_ss_   (mL)** | **RAUC_(0‑τ)_ (%)** | **RC_max_ (%)** |
| 500 mg Q3W  (*n* = 2) | 53,300–98,000 | 207–275 | 47.99 (1.47–94.50) | 62.7–125 | 252.49 (0.00–504.97) | 423–454 | 5.10–9.38 | 3410–5310 | 180–239 | 126–161 |
| 1000 mg Q6W  (*n* = 1) | ND (n = 0) | 472 | 0.53 | ND (n = 0) | ND (n = 0) | 715 | ND (n = 0) | 3680 | ND (n = 0) | 150 |

*AUC* area under the serum dostarlimab concentration-time curve; *C_max_* maximum observed concentration; *DLT-eval* dose-limiting toxicity-evaluable; *GCV%* geometric coefficient of variation; *ND* not determined; *PK/PDy* pharmacokinetics/pharmacodynamics; *RAUC* accumulation ratio compared to cycle 1 data; *RC_max_* maximum observed concentration ratio

^a^Median (range)

^b^*n* = 1

For *n* = 2, only minimum and maximum values are presented; for *n* = 1, only the respective value is presented.

Note that AUC_(0–τ)_ is identical to AUC_(0–last)_ and hence only 1 parameter is presented.

**Supplementary Figure S1** GARNET study design

**
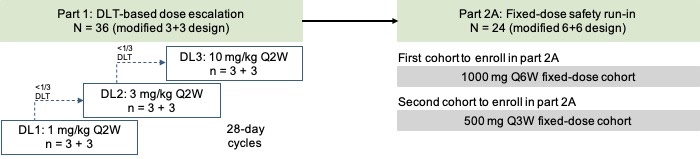
**

*DL* dose level, *DLT* dose-limiting toxicity, *QXW* every X weeks

**Supplementary Figure S2** Patient disposition in the GARNET study


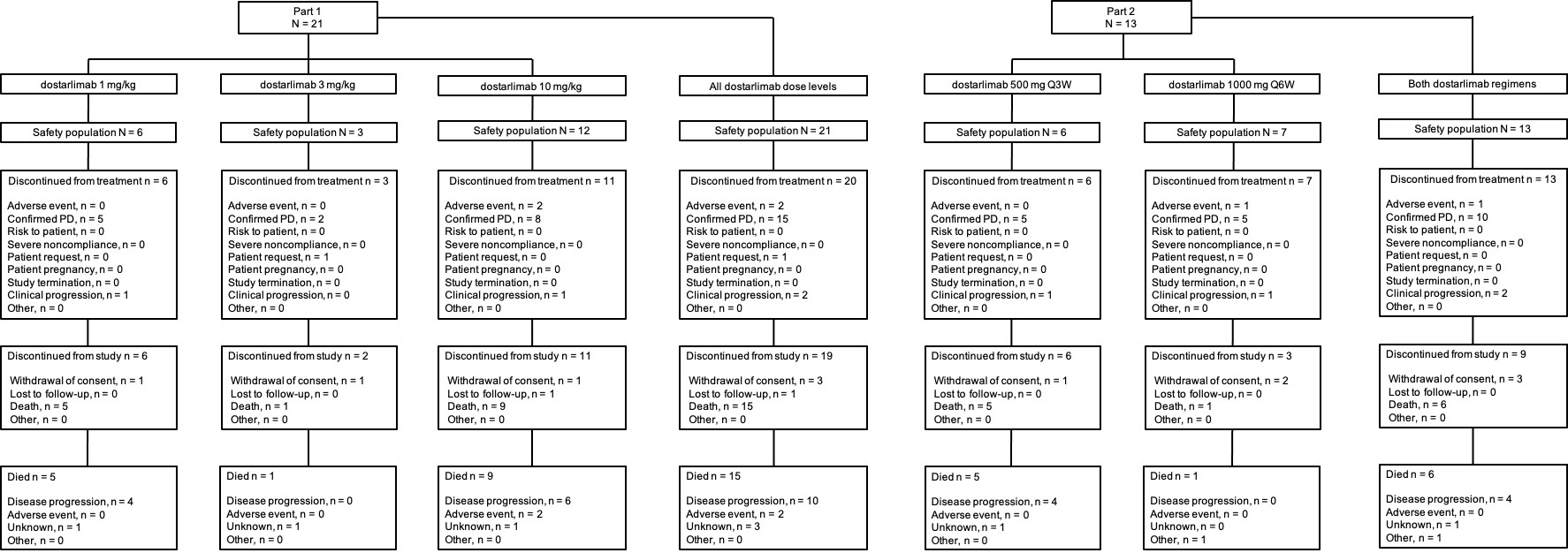


**Supplementary Figure S3** Duration of exposure and overall response in patients with an irCR, irPR, or irSD in parts 1 and 2A by investigator review per irRECIST


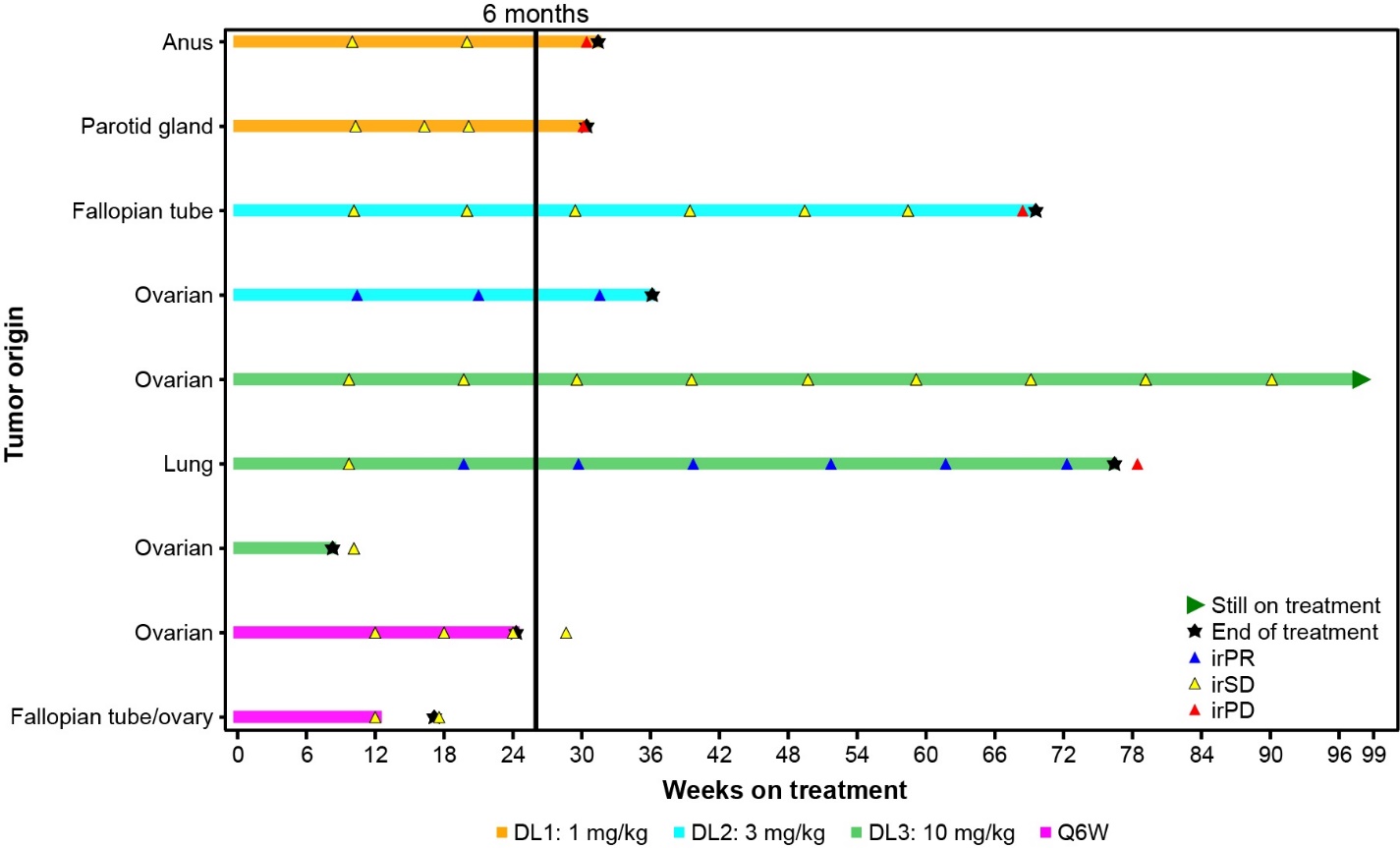


*DL* dose level, *ir* immune-related, *PD* progressive disease, *PR* partial response, *Q6W* every 6 weeks, *SD* stable disease

Each bar represents 1 patient. The length of the bar represents the duration of treatment with dostarlimab.
